# Nature of Beryllium, Magnesium, and Zinc Bonds in Carbene⋯MX_2_ (M = Be, Mg, Zn; X = H, Br) Dimers Revealed by the IQA, ETS-NOCV and LED Methods

**DOI:** 10.3390/ijms232314668

**Published:** 2022-11-24

**Authors:** Filip Sagan, Mariusz Mitoraj, Mirosław Jabłoński

**Affiliations:** 1 Faculty of Chemistry, Jagiellonian University, Gronostajowa 2, 30-387 Cracow, Poland; 2 Faculty of Chemistry, Nicolaus Copernicus University in Toruń, Gagarina 7, 87-100 Toruń, Poland

**Keywords:** carbene, imidazol-2-ylidene, cyclopropenylidene, beryllium bond, magnesium bond, zinc bond, interaction, IQA, ETS-NOCV, LED

## Abstract

The nature of beryllium–, magnesium– and zinc–carbene bonds in the cyclopropenylidene⋯MX${}_{2}$ (M = Be, Mg, Zn; X = H, Br) and imidazol-2-ylidene⋯MBr${}_{2}$ dimers is investigated by the joint use of the topological QTAIM-based IQA decomposition scheme, the molecular orbital-based ETS-NOCV charge and energy decomposition method, and the LED energy decomposition approach based on the state-of-the-art DLPNO-CCSD(T) method. All these methods show that the C⋯M bond strengthens according to the following order: Zn < Mg << Be. Electrostatics is proved to be the dominant bond component, whereas the orbital component is far less important. It is shown that QTAIM/IQA underestimates electrostatic contribution for zinc bonds with respect to both ETS-NOCV and LED schemes. The σ carbene→MX${}_{2}$ donation appears to be much more important than the MX${}_{2}\to$ carbene back-donation of π symmetry. The substitution of hydrogen atoms by bromine (X in MX${}_{2}$) strengthens the metal–carbene bond in all cases. The physical origin of rotational barriers has been unveiled by the ETS-NOCV approach.

## 1. Introduction

Carbenes are undoubtedly one of the most important groups of organic compounds [1,2,3,4,5,6,7,8,9,10,11,12,13,14,15,16,17,18]. They are characterized by having only two valence carbon atoms, thanks to which it generally forms two single covalent bonds to the substituents, CR${}_{1}$R${}_{2}$. As a consequence, the carbon atom retains two unbound electrons, which in a singlet state occupy the same *p* orbital, creating a lone electron pair, which is the cause of nucleophilic properties of carbenes. In other words, singlet carbenes are good Lewis bases. It is therefore not surprising that carbenes participate in various kinds of intermolecular interactions, such as hydrogen bonds [19,20,21,22,23,24,25,26,27], lithium bonds [16,18,28,29,30], beryllium bonds [16,18,31,32,33,34,35], magnesium bonds [16,18,34,35,36,37,38], triel bonds [18,39,40,41,42], tetrel bonds [18,43,44,45], pnictogen bonds [18,46,47,48], chalcogen bonds [18,49], halogen bonds [18,50,51,52,53] (in particular to the iodine atom [50,51]), and aerogen bonds [54]. However, carbenes in particular are known for their great ease of bonding with transition metal atoms [8,12,16,36,55,56,57,58,59,60,61] to form various adducts, which is the goal of numerous studies in organometallic chemistry. In this case, the N-heterocyclic carbenes (NHCs), especially those derived from imidazol-2-ylidene, are of particular importance [4,8,9,11,12,15,16,17,18,27]. In turn, within the group of the NHC–M complexes, those involving zinc are of increasing practical importance [35,36,38,62,63,64,65,66,67,68,69,70,71,72,73,74,75,76,77,78], due in part to the easy availability and relatively low cost of precursors of such complexes. Some practical applications of NHC–Zn complexes in organometallic catalysis have recently been extensively described [62,63,64,65]. It is worth mentioning, however, that apart from the lone electron pair which gives singlet carbenes nucleophilic properties, singlet carbenes also feature electrophilic properties [7,79,80,81,82,83,84] resulting from the presence of an empty *p* orbital perpendicular to the plane formed by CR${}_{1}$R${}_{2}$.

Very recently, one of us (M. J.) has investigated [35] the beryllium [85,86,87,88,89,90,91,92,93], magnesium [85,92,93,94,95,96] and zinc (spodium) [78,97,98,99,100] bonds in a large group of dimers formed by carbenes ((NH${}_{2}$)${}_{2}$C, imidazol-2-ylidene, imidazolidin-2-ylidene, tetrahydropyrymid-2-ylidene, and cyclopropenylidene) or carbodiphosphoranes ((PH${}_{3}$)${}_{2}$C and (NH${}_{3}$)${}_{2}$C) and MX${}_{2}$ (M = Be, Mg, Zn and X = H, F, Cl, Br, Me) molecules. This was the first study of beryllium, magnesium, and zinc bonds of the C⋯M type. Moreover, in the vast majority of cases, in earlier systems with the beryllium or magnesium bond, the Lewis base was a small molecule involving either atom being good electron donors [85,86,87,90,92,93,95] or π-electron bonds [88]. On the other hand, theoretical research on zinc bonds is rather sporadic [35,78,97,100].

Among many important findings was the demonstration that carbene⋯MX${}_{2}$ dimers are characterized by a very high charge transfer from carbene to the MX${}_{2}$ molecule [35]. Namely, it was from two to almost four times greater than in the case of HOH⋯OH${}_{2}$ and HOH⋯NH${}_{3}$ dimers. This result showed that the carbene⋯MX${}_{2}$ (M = Be, Mg, Zn; X = H, F, Cl, Br, Me) dimers are undoubtedly systems in which the charge transfer effect plays a large role, especially in the presence of very polarizable bromine atoms as X. This, in turn, proves the strong interorbital interactions between the carbene molecule and MX${}_{2}$. Importantly, it has been shown that in terms of the electron density and the total electronic energy density values computed at the bond critical point [101] of C⋯M the C⋯Zn zinc bond is similar to the C⋯Be beryllium bond. Treating the value of electron density as a measure of bond strength [101], this result has shown that the zinc bonds should be of similar strength to the beryllium bonds, while the magnesium bonds should be significantly weaker than them [35]. Moreover, together with positive values of the Laplacian of the electron density, negative values of the total electronic energy density obtained for the beryllium and zinc bonds have indicated a significant degree of electron sharing, in turn reflecting a high degree of covalence [102].

A somewhat surprisingly different picture of the similarity of these three types of bonding has been obtained on the basis of the delocalization index, δ(C,M), which describes the exchange of the electrons in the basins of atoms C and M [103,104,105,106]. Namely, the δ(C,M) values for the zinc bond were large, while those for the beryllium and magnesium bond were much lower and similar to each other [35]. Thus, taking into account the relationship of δ(A,B) to the exchange energy [107] and therefore treating δ(A,B) as a measure of the covalent character of an A–B bond [108], this result has shown that the zinc bond is much more covalent than the beryllium and magnesium bond. On the other hand, the NCI-based (the abbreviation NCI is derived from the Non-Covalent Interactions index [109,110]) analysis has shown [35] that the zinc bond is the strongest; however, the beryllium bond is only slightly weaker.

In light of the previously obtained results [35] briefly recalled here, it is necessary to investigate comparatively the physical nature of the beryllium, magnesium and TM-type zinc bonds, not only qualitatively but also quantitatively. This is the main goal of this article, and this nature will be analyzed using complementary energy decomposition methods, IQA (i.e., the Interacting Quantum Atoms approach [111,112]), which provides a local picture of bonding and ETS-NOCV (i.e., the combination of the Extended Transition State (ETS) method [113] with the Natural Orbitals for Chemical Valence (NOCV) method [114,115,116,117,118]), which is more suited for the description of bonding between molecular fragments. Results will also be compared with the state-of-the-art DLPNO-CCSD(T) (i.e., the Domain-based Localized Pair-Natural Orbital Singles and Doubles Coupled Cluster with perturbative Triples) [119,120,121,122,123] calculations and the Local Energy Decomposition (LED) scheme [124,125].

## 2. Results and Discussion

As already mentioned in the Introduction section, one of us (M. J.) has recently investigated [35] beryllium, magnesium and zinc (spodium) bonds in a large group of dimers formed by either a carbene ((NH${}_{2}$)${}_{2}$C, imidazol-2-ylidene, imidazolidin-2-ylidene, tetrahydropyrymid-2-ylidene, and cyclopropenylidene) or a carbodiphosphorane ((PH${}_{3}$)${}_{2}$C and (NH${}_{3}$)${}_{2}$C) and the MX${}_{2}$ (M = Be, Mg, Zn and X = H, F, Cl, Br, Me) species. While the C⋯M contact has been shown to be certainly dominant in all of these dimers, some of them contain additional intermolecular interactions (either hydrogen bond or dihydrogen bond). Since the purpose of this article is to quantify the physical nature of the C⋯M bond, such dimers are not considered here. In other words, in this article, only those dimers in which the described interaction C⋯M is undoubtedly dominant will be considered. The structural characteristics of these dimers will be briefly discussed in the first subsection. Then, in the second and third subsections, the description of the C⋯M interaction obtained by the IQA and ETS-NOCV methods, respectively, will be presented. The correspondence of the results with the outcomes originating from the DLPNO-CCSD(T) method, and based on it, LED energy decomposition will be shown in the fourth subsection. Finally, in the fifth subsection, we will analyze the energy changes of dimers during the rotation of carbene with respect to the ZnBr${}_{2}$ subunit.

### 2.1. Structural Characteristics of the Considered Dimers

**Figure 1:**
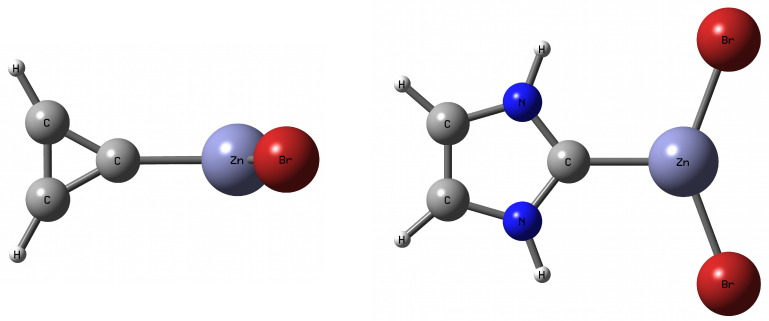
Fully optimized cyclopropenylidene⋯ZnBr${}_{2}$ (**left**) and imidazol-2-ylidene⋯ZnBr${}_{2}$ (**right**) dimer structures.

To meet the aforementioned condition, in this article only the cyclopropenylidene⋯MX${}_{2}$ (M = Be, Mg, Zn; X = H, Br) and, additionally, imidazol-2-ylidene⋯MBr${}_{2}$ dimers will be studied (see Figures S1 and S2). It is worth noting that in the former case, the lack of additional intermolecular interactions (i.e., apart from the described C⋯M) is ensured by the perpendicular orientation of the cyclopropenylidene and MX${}_{2}$ molecules (see the representative cyclopropenylidene⋯ZnBr${}_{2}$ dimer in Figure 1).

In the latter case, however, although the flatness of the entire dimer admits the possibility of the presence of N–H⋯Br hydrogen bonds, these interactions should be relatively weaker than C⋯M due to the large distance H⋯Br and the almost parallel orientation of the N–H and Zn–Br bonds relative to each other (see the representative imidazol-2-ylidene⋯ZnBr${}_{2}$ dimer in Figure 1). A thorough analysis of these intermolecular N-H⋯Br hydrogen bonds will also be performed. The values of some fundamental parameters characterizing the considered carbene⋯MX${}_{2}$ dimers are presented in Table 1.

As can be seen from Figure 1 and Table 1 (see the $\alpha_{\mathrm{XMX}}$ angle), the characteristic feature of the considered dimers is the clearly bent structure of the MX${}_{2}$ molecule. It is worth noting that this bend is similar in the case of MX${}_{2}$ molecules with beryllium or zinc, while it is clearly smaller in the case of the magnesium atom, which forms more ionic and thus more resistant M–X bonds. Thus, the value of the angle $\alpha_{\mathrm{XMX}}$ suggests the similarity between the Be and Zn atoms. It should be noted that the bending of the MX${}_{2}$ molecule is a characteristic effect of the presence of a beryllium or magnesium bond in dimers with different Lewis bases [85,86,88,92]. Moreover, Martín-Sómer et al. [86] have shown that in the case of H${}_{3}$N⋯BeH${}_{2-n}$X${}_{n}$ (X = F, Cl, Br; n≤ 2) dimers, the nonlinearity of the BeH${}_{2-n}$X${}_{n}$ molecule is due to the decrease in LUMO energy. It is also worth noting that for the bond length M–X the relation Be<Zn<Mg applies so that for a given X the magnesium atom forms the longest M–X bond. It should also be emphasized that the formation of the C⋯M bond leads to significant elongation of the M–X bond (see $\Delta d_{\mathrm{MX}}$ in Table 1) and a greater opening of the L–C–L angle in the carbene molecule (see $\Delta \alpha_{\mathrm{LCL}}$ in Table 1). For the same MX${}_{2}$ molecule, both of these effects are clearly greater in imidazol-2-ylidene than in cyclopropenylidene, suggesting that the C⋯M interaction should be stronger in the dimers of imidazol-2-ylidene. This is confirmed by the computed dissociation energies [35]. For imidazol-2-ylidene, these energies are in the 41.2–48.6 kcal/mol range, while for cyclopropenylidene, only 28.2–35.4 kcal/mol (for the same set of MX${}_{2}$ molecules, i.e., MBr${}_{2}$), similar outcomes are valid when considering other XC and DLPNO-CCSD(T) method, Table S1. The greatest dissociation energy value characterizes the dimers with Be, while the smallest $D_{0}$ are noted in the dimers containing Zn, which can only partially be explained by the shortest distance C⋯M in the case when M = Be. This is because in the case of Mg and Zn, the distance C⋯Mg is longer than that of C⋯Zn, but the magnesium bond is stronger than the zinc bond. For example, for the cyclopropenylidene⋯MH${}_{2}$ dimers, the C⋯M lengths for Be, Mg, and Zn, respectively, are 1.743, 2.268, and 2.121 Å, while the dissociation energies are 29.1, 20.9, and 15.2 kcal/mol. This result shows that the dissociation energy is not completely dependent on the distance C⋯M. Such a trend has already been observed in literature [35].

It has already been mentioned in Introduction that the carbene⋯MX${}_{2}$ dimers considered here are characterized by a very high charge transfer effect from the Lewis base, i.e., the carbene molecule, to the Lewis acid, i.e., the MX${}_{2}$ molecule, and this effect is particularly large in the presence of highly polarizable bromine atoms. Indeed, in the case of the imidazol-2-ylidene⋯BeBr${}_{2}$ dimer, the value of the charge transfer (based on chemically reliable [128,129,130] Hirshfeld atomic charges [126,127]) is as high as −0.464 au. It is enough to mention that the charge transfer determined on the same level of theory in the case of the water dimer is only −0.098 au. This comparison suggests the presence of strong interorbital interactions between the carbene molecule and the MX${}_{2}$ unit. Their significance, however, can be quantified by ETS-NOCV and LED methods.

### 2.2. IQA-Based Results

As already mentioned in the Methods section, the IQA approach is a unique method that enables the determination of the interaction energy of any two atoms and the decomposition of this energy into individual terms, according to Equations (1) and (2). The results of such decomposition carried out for the contact C⋯M are shown in Table 2.

A certain inconvenience of IQA (which is also a characteristic feature of energy decomposition methods) is that the components of the interaction energy are generally much larger than the interaction energy itself. This is obviously due to the close cancellation of the negative and largest $E_{\mathrm{neen}}$ and the positive sum of $E_{\mathrm{nn}}$ and $E_{\mathrm{ee},C}$. It is obvious that the rapid growth of the relevant components with the change Be→Mg→Zn results from both the increase in the atomic number of M, i.e., the charge of the atomic nucleus, and the increase in the number of electrons. However, it is much more important that according to IQA, the C⋯M (M = Be, Mg, Zn) interaction in all the dimers with the participation of cyclopropenylidene is stabilizing, which is indicated by negative values of the obtained interaction energies. For the same X, the binding effect increases in the Zn→Mg→Be direction and is slightly stronger when X = Br, so that the strongest C⋯M bond is for BeBr${}_{2}$ (−0.3644 a.u.).

It is interesting to look at the values of the exchange-correlation energy ($E_{\mathrm{ee},\mathrm{xc}}$) as well as the percentage share of this component in the total interaction energy (%$E_{\mathrm{ee},\mathrm{xc}}$). Of course, independently of X, $E_{\mathrm{ee},\mathrm{xc}}$ increases (i.e., becomes more negative) in the direction Mg→Be→Zn, with the corresponding values being slightly greater for Br than for H. Importantly, the values for Zn are significantly greater than those for Be or Mg (e.g., $E_{\mathrm{ee},\mathrm{xc}}$ is −0.1091 a.u. in cyclopropenylidene⋯ZnH${}_{2}$ and only −0.0458 a.u. and −0.0307 a.u. in cyclopropenylidene⋯BeH${}_{2}$ and cyclopropenylidene⋯MgH${}_{2}$, respectively). The contribution of negative electrostatic energy ($E_{\mathrm{elst}}$) in the case of the cyclopropenylidene dimers quickly decreases in the Be→Mg→Zn series, as a consequence of which the percentage of the exchange-correlation energy quickly increases from ca. 12–13% for the beryllium bond, to ca. 16% for the magnesium bond and up to ca. 63–66% for the zinc bond. This result shows that the IQA method suggests that the zinc bond is highly covalent, whereas the beryllium and magnesium bonds are electrostatic in nature. Of course, such a result was to be expected, because, as already mentioned in the Introduction section, the same trend has already been obtained earlier for the delocalization index δ(C,M) [35], which, as is known [107,108], correlates very well with the exchange-correlation energy.

Interestingly, IQA has given quite small interaction energies for the imidazol-2-ylidene⋯MBr${}_{2}$ dimers, which do not match the significant dissociation energies for these complexes; Table 1. Even more surprisingly, a positive interaction energy value (0.0379 a.u.) has been obtained for the imidazol-2-ylidene⋯MgBr${}_{2}$ dimer. This destabilizing effect of C⋯Mg (0.0379 a.u.) is due to a significant destabilizing electrostatic contribution (0.0777 a.u.), which exceeds the negative exchange-correlation energy (−0.0397, i.e., ca. 51% only). In the case of imidazol-2-ylidene⋯ZnBr${}_{2}$, $E_{\mathrm{elst}}$ is also positive (0.1190 a.u.; and therefore the purely Coulombic contribution gives no bonding), but unlike for imidazol-2-ylidene⋯MgBr${}_{2}$, this contribution is excessively overshadowed (over 110%) by $E_{\mathrm{ee},\mathrm{xc}}$ (−0.1317 a.u.), which gives stabilizing C⋯Zn bond, which could be considered as anti-electrostatic and covalent.

These diatomic IQA data do not explain the stability of the imidazol-2-ylidene⋯MgBr${}_{2}$ species. To this end, we have also summed the interaction energies corresponding to the pairs of atoms in the groups representing the {carbene}⋯{MX${}_{2}$} fragmentation (i.e., all the combinations where one of the atoms is in the {carbene} moiety and the other one in {MX${}_{2}$}). In such treatment, which in fact ignores many-body effects, all the $E_{\mathrm{int}}$ values are negative, as expected. Furthermore, the IQA fragment-based data (see Table 3) indicate domination of $E_{\mathrm{elst}}$ over $E_{\mathrm{xc}}$ for the Be- and Mg-based systems ( ca. 51–58% of E${}_{\mathrm{int}}$), with the Zn-based species as the only exceptions and the share of electrostatics in their total stabilization reaching ca. 16–21% only.

For the imidazol-2-ylidene complexes, among all the {carbene}⋯{MX${}_{2}$} atomic pairs, the most stabilizing according to IQA are N⋯M interactions, providing from ca. −150 kcal/mol in the case of Zn via. ca. −220 kcal/mol (Mg) to ca. −250 kcal/mol (Be), followed by C⋯Br (from ca. −43 kcal/mol to ca. −39 kcal/mol) and H⋯Br (from ca. −48 kcal/mol to ca. −32 kcal/mol), Table S2. Surprisingly, the C⋯M interactions are not among the most stabilizing ones, ranging from +20 kcal/mol (Mg) to −40 kcal/mol (Be). In the case of cyclopropenylidene complexes, the C⋯M interactions are definitely the most important, ranging from ca. −100 to over −200 kcal/mol, Tables S3 and S4.

In the fragment-based treatment, a quite different picture of the C⋯M bond nature has been obtained. Namely, for cyclopropenylidene⋯MX${}_{2}$, the exchange-correlation energy component became more prominent (ca. 45% for Be and Mg, and up to 80% in the case of Zn; Table 3). Concurrently, in the fragment-based approach, all the MX${}_{2}$ complexes with imidazol-2-ylidene have negative $E_{\mathrm{int}}$ values, as well as their electrostatic and exchange-correlation components; Table 3. Moreover, the character of these interactions changed in the case of Be and Mg as the electrostatic energy became dominant.

Clearly, the fragment-based approach seems to be much more credible for the description of the carbene⋯metal bonds in both cyclopropenylidene and imidazol-2-ylidene complexes. However, the lack of the C⋯M interaction among the most important contributions is alarming, as much as the counterintuitive positive $E_{\mathrm{int}}$ value for the imidazol-2-ylidene⋯MgBr${}_{2}$ dimer (Table 2). The most probable explanation is a quite significant positive QTAIM charge on the metal atoms (e.g., the QTAIM-based atomic charge of Mg is as much as 1.687 a.u. compared to 0.616 a.u. only obtained from the Hirshfeld method) [35]. This, coupled with the incorrect sign of charges on the carbene carbon atom (already stressed in the first work concerning these systems [35]), puts the reliability of IQA electrostatics in question. Since the IQA method clearly depends on the charge distribution between QTAIM atomic basins and therefore provides variable outcomes for different charge density partitionings, we will switch to ETS-NOCV, which estimates $E_{\mathrm{elst}}$ in a more reliable way through the quasi-classical approach based on self-consistently converged fragment electron densities; see Methods.

### 2.3. ETS-NOCV-Based Results

In this section, we will present the results obtained by means of the molecular orbital-based ETS-NOCV method. We have used singlet {carbene} and {MX${}_{2}$} fragments which have appeared to be more suitable than triplets since they deliver the smallest orbital interaction contribution. The obtained values of the ETS-NOCV energy terms (see Equation (5)) are listed in Table 4.

Contrary to the atomic resolution-based IQA results, all ETS-NOCV interaction energies ($\Delta E_{\mathrm{int}}$) are negative and correspond better to the bond dissociation energies ($D_{0}$ values in Table 1). Bonds formed by beryllium are the strongest, followed by energetically similar magnesium and zinc bonds. Systems with Br atoms are bonded noticeably stronger than with H atoms, and complexes with imidazol-2-ylidene are stabilized more than their cyclopropenylidene counterparts. The carbene⋯metal bonds are dominated by electrostatics ($\Delta E_{\mathrm{elst}}$, Table 4), with its percentage contribution to the total stabilization being 67–71% in the case of Mg and Zn dimers and 56–61% in systems with Be. This is similar to other reports of bonding in transition metal complexes of imidazol-2-ylidene-based NHCs [12,56,57,58,59,61] and other model carbenes [55,58,61]. For example, it turned out that the electrostatic contribution to C⋯M bonds in imidazol-2-ylidene complexes with Cu, Ag and Au halides accounts for more than 65% of the binding energy [56]. This contribution was also very similar (ca. 2/3 of the interaction energy) in the various NHC–TM complexes studied by Tonner et al. [58]. It is worth mentioning at this point the excellent review on the NHC–M bond by Jacobsen et al. [12], in which it was found that NHC–M bonds are mainly electrostatic in origin with a percentage contribution from 60% to 80%.

**Figure 2:**
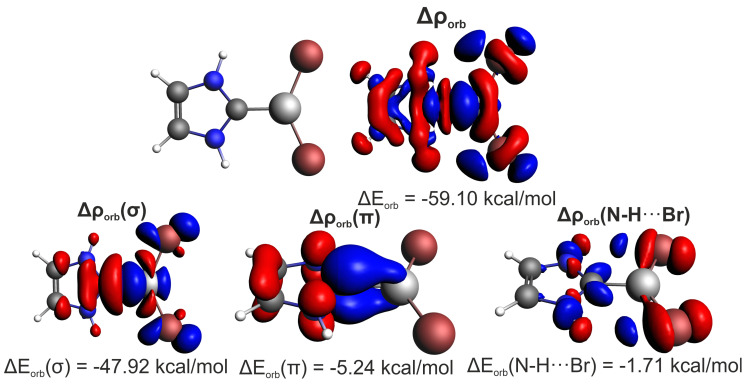
Dominant NOCV-based deformation density contributions determined for imidazol-2-ylidene⋯BeBr${}_{2}$ dimer.

Orbital interaction is the second strongest energy term, reaching up to 40% of total stabilization in beryllium systems and 25–30% in the case of Mg and Zn systems, Table 4. The carbene→metal donation (the LP(C)$\to \sigma^{☆}$(MX) transfer in MO language) dominates by far (72–87%) over the metal→carbene back-donation in π symmetry, with 16–20% contribution in the case of systems with X = H and 7–12% for X = Br. The clearly lower back-donation effect is visible in the latter case, resulting from the electron-withdrawing properties of Br atoms. It should be pointed out that in many transition metal complexes, the π-back-bonding may be far more pronounced and can reach even 40% of the overall orbital interaction energy [60]. Finally, it should be commented that the geometry distortion term appeared to be the most pronounced for Be-species, Table S5. Also noteworthy, weak (up to −1.7 kcal/mol, i.e., ca. 3% of the orbital interaction energy) side N–H⋯Br hydrogen bonds have been identified in the imidazol-2-ylidene⋯MBr${}_{2}$ complexes, Figure 2.

Such a negligible share of N–H⋯Br hydrogen bonds really confirms the correct choice of systems with the dominant C⋯M interaction. As ETS-NOCV is only capable of separating the $\Delta E_{\mathrm{orb}}$ contribution, it is necessary to support it with complementary IQA results, which indicate a strong electrostatic component ($E_{\mathrm{elst}}^{H\cdots \mathrm{Br}}$ represents 95% of $E_{\mathrm{int}}^{H\cdots \mathrm{Br}}$ amounting to −45.5 kcal/mol). Such pronounced interaction energies are common for a two-atomic approach. For example, for the hydrogen bond in water, the ΔE${}_{\mathrm{int}}^{O\cdots H}$ value amounts to ca. −100 kcal/mol [111].

### 2.4. LED-Based Results

Local Energy Decomposition [124,125] allows for partitioning of the binding energy at the DLPNO-CCSD(T) [120,121,122] level of theory, often considered as the `gold standard’ for calculating interaction energies. The same fragments as before, i.e., {carbene} and {MX${}_{2}$}, have been chosen. The binding energies obtained at the DLPNO-CCSD(T) level of theory are consistent with the DFT-based energies, that is, the beryllium bonds are the strongest, followed by magnesium and zinc (spodium) bonds; compare Table 1 and Table 5.

Local Energy Decomposition partitioning confirms the importance of electrostatics in metal–carbene bonding, with E${}_{\mathrm{elst}}^{\mathrm{ref}}\approx$ 90% of total stabilization for all compounds, Table 5. Dispersion contribution of ca. 2% of the total interaction energy proved to be very small. The charge-transfer contributions, bottom of the Table 5, confirm qualitatively the picture obtained by ETS-NOCV, i.e., the carbene → MX${}_{2}$ donation being far more important than MX${}_{2}\to$ carbene back–donation.

### 2.5. Flat vs. Perpendicular Structure of the Carben⋯MX${}_{2}$ Dimer

**Figure 3:**
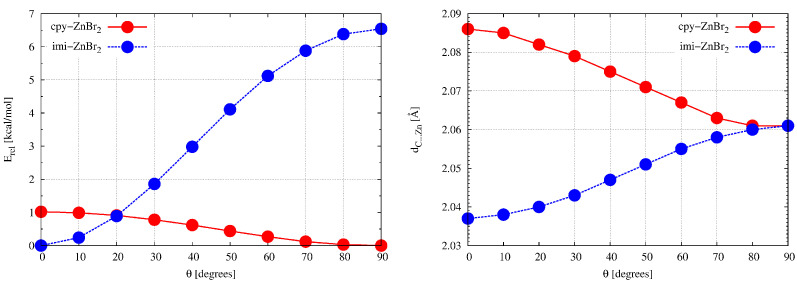
Relation between the relative energy (i.e., determined in relation to the minimum for a given dimer) (left) or the distance $d_{C\cdots \mathrm{Zn}}$ (right) and the twist angle of the ZnBr${}_{2}$ unit plane with respect to the carbene plane for dimers cyclopropenylidene⋯ZnBr${}_{2}$ (cpy-ZnBr${}_{2}$) and imidazol-2-ylidene⋯ZnBr${}_{2}$ (imi-ZnBr${}_{2}$).

Looking at Figure 1, showing the cyclopropenylidene ⋯ZnBr${}_{2}$ and imidazol-2-ylidene⋯ZnBr${}_{2}$ dimers, the fundamental difference between them in the orientation of the ZnBr${}_{2}$ molecule relative to the carbene plane is clearly visible. Namely, the ZnBr${}_{2}$ and carbene units are perpendicular to each other in cyclopropenylidene⋯ZnBr${}_{2}$, while imidazol-2-ylidene⋯ZnBr${}_{2}$ is planar. Taking into account the fact that the cause of the twisting of the metal-unit in relation to the carbene plane was described in the literature only very sporadically [78], and taking advantage of the relatively high structural simplicity of the studied systems, it was tempting to investigate the cause of the structural differences regarding the twisting of the MX${}_{2}$ and carbene planes in relation to each other. For this purpose, we have performed additional calculations for the twisted structures of both dimers, i.e., cyclopropenylidene⋯ZnBr${}_{2}$ and imidazol-2-ylidene⋯ZnBr${}_{2}$, in which the twist angle (denoted by θ) L-C-Zn-Br (L is either C or N) was changed every 10${}^{\circ }$ from 0${}^{\circ }$ to 90${}^{\circ }$. Figure 3 (left) shows the energy profile related to the change in θ from 0${}^{\circ }$ to 90${}^{\circ }$.

It is clearly visible that although the equilibrium geometry of the cyclopropenylidene ⋯ZnBr${}_{2}$ dimer corresponds to the perpendicular structure (i.e., with θ = 90${}^{\circ }$), the planar structure (θ = 0${}^{\circ }$) is energetically less stable by only 1 kcal/mol so that the rotation of the ZnBr${}_{2}$ unit around the C⋯Zn bond axis is practically free, Figure 3. Dimer imidazol-2-ylidene⋯ZnBr${}_{2}$ presents a completely different situation, namely the flat structure of this dimer is more stable than the perpendicular structure by about 6.5 kcal/mol which proves hindered rotation of ZnBr${}_{2}$ due to the presence of adjacent N–H bonds, Figure 1. Planarization clearly favors the formation of residual hydrogen bonds N–H⋯Br giving rise to Zn⋯C bond contraction by ca. 0.023 Å; Figure 3, right.

**Figure 4:**
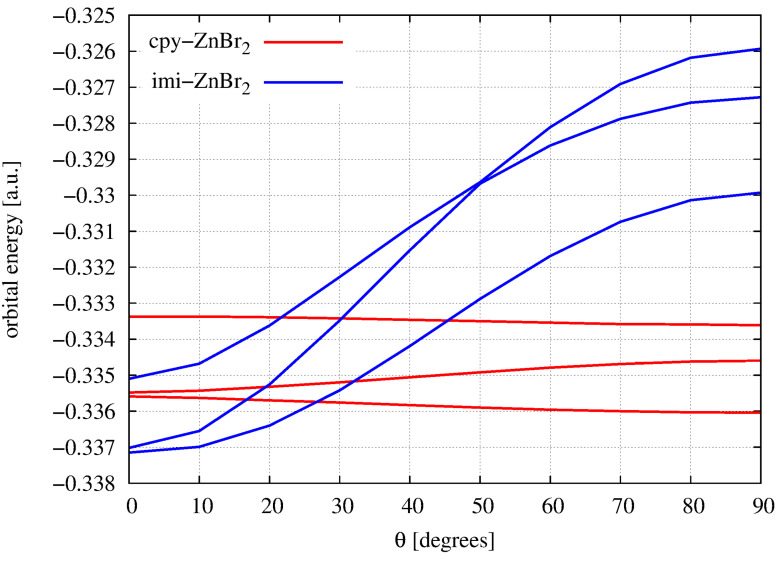
Dependence of the orbital energy of the three highest occupied orbitals (i.e., HOMO, HOMO-1 and HOMO-2) on the twist angle of the ZnBr${}_{2}$ unit plane with respect to the carbene plane for dimers cyclopropenylidene⋯ZnBr${}_{2}$ (cpy-ZnBr${}_{2}$) and imidazol-2-ylidene⋯ZnBr${}_{2}$ (imi-ZnBr${}_{2}$).

According to the Walsh rule [131], a molecule adopts the conformation that most ensures stabilization of HOMO. A Walsh diagram showing the dependence of the orbital energies of the three highest occupied orbitals, i.e., HOMO, HOMO-1 and HOMO-2, on the twist angle θ is shown in Figure 4.

It is clearly visible that in the case of imi-ZnBr${}_{2}$, i.e., the imidazol-2-yliden⋯ZnBr${}_{2}$ complex, the 90${}^{\circ }\to$ 0${}^{\circ }$ rotation leads to a greater stabilization of all the orbitals considered, and thus also of HOMO. Thus, based on Walsh’s rule, the planar structure should be more stable for this complex than the perpendicular structure, which is indeed true (see Figure 1 and Figure 3). By the way, it is worth noting that both structures are characterized by reversed positions of HOMO and HOMO-1. In the case of the cyclopropenylidene⋯ZnBr${}_{2}$ dimer, the 0${}^{\circ }\to$ 90${}^{\circ }$ rotation leads to the stabilization of HOMO, but this stabilization is so minimal that it is doubtful to see this stabilization as the cause of the perpendicular structure of this dimer (Figure 1). Nevertheless, small changes in orbital energies may perhaps explain a small change in total energy (Figure 3) and almost unhindered rotation around the C⋯Zn axis.

**Figure 5:**
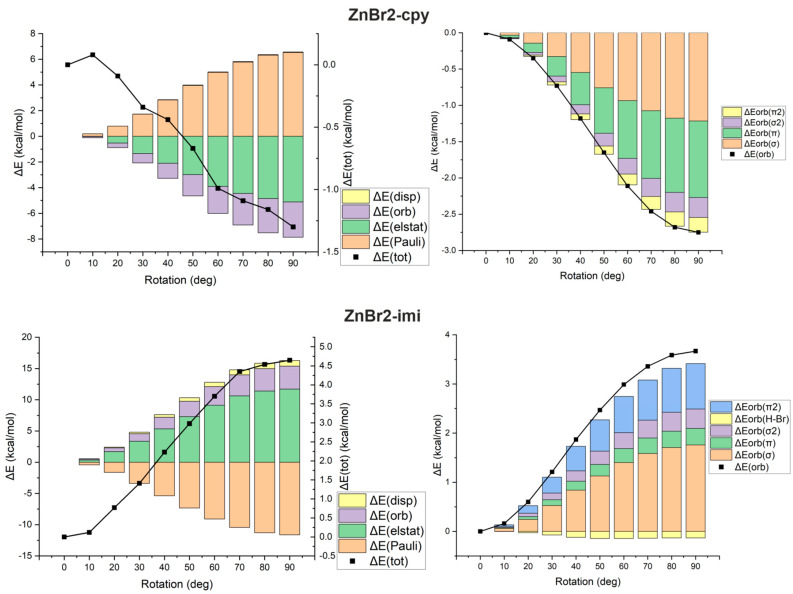
Changes in the values of the ETS-NOCV-based interaction energy components (left) or the orbital energy components (right) with the change of the rotation angle of the ZnBr${}_{2}$ unit plane with respect to the carbene plane for dimers cyclopropenylidene⋯ZnBr${}_{2}$ (changes relative to θ = 0${}^{\circ }$; ZnBr${}_{2}$-cpy) and imidazol-2-ylidene⋯ZnBr${}_{2}$ (changes relative to θ = 90${}^{\circ }$; ZnBr${}_{2}$-imi).

However, in order to make a more reliable assessment of structural changes, ETS-NOCV contributions along rotation angle have been determined and depicted in Figure 5.

It can be seen that the significant shortening (0.024 Å) of the C⋯Zn distance upon planarization of the imidazol-2-ylidene⋯ZnBr${}_{2}$ dimer (see Figure 3, right) is due to an increase in the orbital interaction term and, to a larger extent, the electrostatic contributions, which causes a drop of $\Delta E_{\mathrm{int}}$ by ca. 4.7 kcal/mol. The former, i.e., the orbital interactions, results mainly from the amplification of the σ(Zn–C) component and, to a lesser extent, the π(Zn–C) component, Figure 5. Interestingly, the effect of twisting (0${}^{\circ }\to$ 90${}^{\circ }$) the cyclopropenylidene⋯ZnBr${}_{2}$ dimer also causes a similar shortening (0.025 Å) of the distance C⋯Zn (Figure 3, right). Nevertheless, the overall C⋯Zn interaction energy ($\Delta E_{\mathrm{int}}$) changes only slightly by ca. 1.3 kcal/mol, which is consistent with a facile rotation. Such small variations in C⋯Zn bond interaction energies are caused by modifications of both orbital interaction and electrostatic contributions, Figure 5. Finally, it is worth mentioning that rotation around the C⋯Zn axis in the cyclopropenylidene⋯ZnBr${}_{2}$ dimer has practically no effect on the Zn–Br bond length, while in the imidazol-2-ylidene⋯ZnBr${}_{2}$ dimer the transition from perpendicular to planar structure slightly lengthens the bond by 0.006 Å. This effect can be attributed to a larger LP(C) $\to \sigma^{*}$(Zn–Br) donation.

## 3. Methods and Materials

Geometries of monomers and dimers were fully optimized on the ωB97X-D/6-311++G (2df,2p) level of theory, that is utilizing the ωB97X-D range-separated hybrid GGA exchange-correlation functional [132] of Density Functional Theory (DFT) [133,134,135,136] and the 6-311++G(2df,2p) basis set [137,138,139,140,141,142,143], which includes both polarization and diffuse functions. Mardirossian and Head-Gordon have shown [136] that ωB97X-D is one of the best exchange-correlation functionals among 200 for general purposes including intermolecular interactions. To increase the accuracy of the optimization procedure and numerical integration, cutoffs on forces and step size that are used to determine convergence were additionally tightened (0.000015 and 0.000010 for maximum force and its root mean square, respectively, and 0.000060 and 0.000040 for maximum displacement and its root mean square, respectively) and integration grid was increased to the (99, 590) one (UltraFine) having 99 radial shells and 590 angular points per shell. Vibration analysis showed that all the considered systems correspond to real minima on potential energy surfaces. Both geometry optimization and vibration analysis were performed by means of Gaussian 09 [144].

The Interacting Quantum Atoms (IQA) approach [111,112], which is based on Bader’s QTAIM (i.e., Quantum Theory of Atoms in Molecules) [101], allows the total energy of a system to be divided into mono- and polyatomic contributions. Of the many useful terms defined by the IQA method, this article will utilize the interatomic interaction energy:

(1) $$E_{\mathrm{int}}^{E_{1}E_{2}}=E_{\mathrm{nn}}^{E_{1}E_{2}}+E_{\mathrm{ne}}^{E_{1}E_{2}}+E_{\mathrm{en}}^{E_{1}E_{2}}+E_{\mathrm{ee}}^{E_{1}E_{2}}\;\;\;\;\;\;\left(E_{1}\neq E_{2}\right)$$

where $E_{\mathrm{nn}}^{E_{1}E_{2}}$ is the repulsion energy between nuclei of atoms E${}_{1}$ and E${}_{2}$, $V_{\mathrm{ne}}^{E_{1}E_{2}}$ is the attraction energy between the nucleus of the atom E${}_{1}$ and the electrons of the atom E${}_{2}$, $E_{\mathrm{en}}^{E_{1}E_{2}}$ is the attraction energy between electrons of the atom E${}_{1}$ and the nucleus of the atom E${}_{2}$ and $E_{\mathrm{ee}}^{E_{1}E_{2}}$ is the interatomic two-electron repulsion energy. The sum of the middle two terms gives the energy of the interatomic nucleus–electron attraction ($E_{\mathrm{neen}}^{E_{1}E_{2}}$). Importantly, the interelectron repulsion energy can be further divided into a sum of the purely classical (Coulombic) contribution and the exchange-correlation (i.e., the non-classical term) energy:

(2) $$E_{\mathrm{ee}}^{E_{1}E_{2}}=E_{\mathrm{ee},C}^{E_{1}E_{2}}+E_{\mathrm{ee},\mathrm{xc}}^{E_{1}E_{2}}$$

Moreover, the sum of the first three terms in Equation (1) and the classical interelectron energy contribution gives the electrostatic energy, such that:

(3) $$E_{\mathrm{int}}^{E_{1}E_{2}}=E_{\mathrm{elst}}^{E_{1}E_{2}}+E_{\mathrm{ee},\mathrm{xc}}^{E_{1}E_{2}}$$

As one can see, all the terms in the IQA equations have strictly defined physical meanings. It is also worth emphasizing that an extremely valuable feature of the IQA decomposition method is the fact that it does not require any reference system and that the interatomic interaction energy can be determined for any pair of E${}_{1}$ and E${}_{2}$ atoms, e.g., not necessarily connected with each other by a bond path. In this article, the interatomic interaction energy and its components will be determined for the C⋯M (M = Be, Mg, Zn) interaction. Moreover, for an estimation of the total interaction energy between carbene and metal moieties, pair atomic energies between all atoms in both fragments were summed, such as for moieties G and H:

(4) $$E_{\mathrm{int}}^{\mathrm{GH}}=\sum_{A\in G}\sum_{B\in H}E_{\mathrm{int}}^{\mathrm{AB}}$$

The AIMAll program [145] was utilized for this purpose. MP2/6-311+G(d,p)-based [146,147] wave functions generated in Gaussian09 were used based on the aforementioned geometries.

The ETS-NOCV method [118] is a combination of the ETS Ziegler–Rauk method [113] with the Natural Orbitals for Chemical Valence (NOCV) method [114,115,116,117]. In the ETS method, the total interaction energy between fragments A and B (atoms, fragments and molecules) is decomposed according to the following equation:

(5) $$\Delta E_{\mathrm{total}}=\Delta E_{\mathrm{dist}}+\Delta E_{\mathrm{elst}}+\Delta E_{\mathrm{Pauli}}+\Delta E_{\mathrm{disp}}+\Delta E_{\mathrm{orb}}=\Delta E_{\mathrm{dist}}+\Delta E_{\mathrm{int}}$$

where $\Delta E_{\mathrm{dist}}$ is the distortion energy that is needed to transform the structures of the isolated fragments A and B into the geometries they have in the created A–B system, $\Delta E_{\mathrm{elst}}$ is the energy of the electrostatic interaction of fragments A and B (in their final positions in the molecule, but frozen electron densities), $\Delta E_{\mathrm{Pauli}}$ is the Pauli repulsion energy resulting from the repulsion between electrons with same spins of fragments A and B, $\Delta E_{\mathrm{disp}}$ is the dispersion energy, and $\Delta E_{\mathrm{orb}}$ is the orbital interaction energy associated with the formation of the A–B system (e.g., an A–B bond) and results from the interaction between the occupied orbitals of the A subsystem with the virtual orbitals of the B subsystem and vice versa. This term also contains the polarization energy related to the electron density changes in each of the A and B subsystems and the interfragment charge transfer. The last four terms of Equation (5) form the interaction energy, $\Delta E_{\mathrm{int}}$. On the other hand, the NOCV orbitals are eigenvectors of the differential charge and bond orders matrix, $\Delta P=P-P^{0}$, where P is the density matrix of a molecule (e.g., A–B), and $P^{0}$ is the sum of the density matrices of the orthogonalized fragments A and B, $P_{A}^{0}+P_{B}^{0}$. The overriding feature of the NOCV orbitals representation is the ability to decompose the differential electron density, $\Delta \rho_{\mathrm{orb}}=\rho_{\mathrm{AB}}-\rho_{A}^{0}-\rho_{B}^{0}$, into chemically meaningful $\Delta \rho_{\mathrm{orb}}^{k}$ diagonal contributions:

(6) $$\Delta \rho_{\mathrm{orb}}=\sum_{k=1}^{M/2}\nu_{k}\left[-\psi_{-k}^{2}+\psi_{k}^{2}\right]=\sum_{k=1}^{M/2}\Delta \rho_{\mathrm{orb}}^{k}$$

where $\nu_{k}$ are the eigenvalues obtained by diagonalizing the matrix ΔP, and *M* is the number of basis set functions. Visualization of contributions $\Delta \rho_{\mathrm{orb}}^{k}$ enables the identification and characterization of the individual components (σ, π, δ, etc.) of a bond. Importantly, under the ETS-NOCV method, it is possible to quantify the energies ($\Delta E_{\mathrm{orb}}^{k}$) corresponding to the individual $\Delta \rho_{\mathrm{orb}}^{k}$ components:

(7) $$\Delta E_{\mathrm{orb}}=\sum_{k=1}^{M/2}\nu_{k}\left[-F_{-k,-k}^{\mathrm{TS}}+F_{k,k}^{\mathrm{TS}}\right]=\sum_{k=1}^{M/2}\Delta E_{\mathrm{orb}}^{k}$$

Calculations under the ETS-NOCV method were performed on the BLYP-D3(BJ) [148,149,150]/ TZP-ZORA [151,152] level of theory using the ADF program [153]. Such a computational protocol proved to be adequate for bonding analysis both in our and other previous studies [154,155]. In this work, we will focus on the instantaneous interaction energy $\Delta E_{\mathrm{int}}$, since total energy has already been studied in Ref. [35].

The Domain-based Localized Pair-Natural Orbital Singles and Doubles Coupled Cluster with perturbative Triples [120,121,122,123] (DLPNO-CCSD(T)) approach is a relatively new approximation of the Coupled Clusters method, which in turn is often referred to as a `golden standard’ for quantum-chemical calculations [142]. DLPNO-CCSD(T) itself recovers >95% of triples contribution and 99.8% of correlation energy, and thanks to its scaling being close to DFT, it is the first CCSD(T) approximation capable of calculating the entire protein [122]. It is performed by localizing the orbitals and generating the Projected Atomic Orbitals from occupied localized orbitals, followed by creation of Pair Natural Orbitals from selected PAOs [119,120].

Local Energy Decomposition [124,125] was created for the DLPNO-CCSD(T) approach in order to harness its accuracy in determining interaction energies and provide an energy decomposition scheme on the CCSD(T) level of theory. It decomposes the binding energy into the following quantities:

(8) $$\Delta E=\Delta E_{\mathrm{geo}-\mathrm{prep}}+\Delta E_{\mathrm{int}}$$

The first component is the energy needed to distort fragments A and B from their optimal geometries to their final state in the AB adduct. On the other hand, $\Delta E_{\mathrm{int}}$ is the interaction energy of said fragments, which can be further decomposed into reference (HF) energy, $\Delta E_{\mathrm{int}}^{\mathrm{HF}}$, and a correlation contribution ($\Delta E_{\mathrm{int}}^{C}$):

(9) $$\Delta E_{\mathrm{int}}=\Delta E_{\mathrm{int}}^{\mathrm{HF}}+\Delta E_{\mathrm{int}}^{C}$$

The Hartree–Fock part can in turn be expanded into:

(10) $$\Delta E_{\mathrm{int}}^{\mathrm{HF}}=\Delta E_{\mathrm{el}-\mathrm{prep}}^{\mathrm{HF}}+\Delta E_{\mathrm{elstat}}+\Delta E_{\mathrm{exch}}$$

where $\Delta E_{\mathrm{el}-\mathrm{prep}}^{\mathrm{HF}}$ is the energy needed to distort the electronic structure of fragments into the state optimal for fragment interaction. Electrostatic interaction between monomers is denoted by $\Delta E_{\mathrm{elstat}}$, and the HF exchange by $\Delta E_{\mathrm{exch}}$. Correlation energy is decomposed into:

(11) $$\Delta E_{\mathrm{int}}^{C}=\Delta E_{\mathrm{no}-\mathrm{disp}}^{C}+\Delta E_{\mathrm{disp}}^{C}+\Delta E_{\mathrm{int}}^{C-(T)}$$

Terms $\Delta E_{\mathrm{disp}}^{C}$ and $\Delta E_{\mathrm{no}-\mathrm{disp}}^{C}$ are correlation energy contributions associated with dispersion and non-dispersive corrections, respectively. The term denoted by $\Delta E_{\mathrm{int}}^{C-(T)}$ is the triples correction arising from CCSD(T).

## 4. Conclusions

This article presents a comparative study of a series of metal–carbene bonds between main- and transition group metals: beryllium, magnesium and zinc, and model carbenes: cyclopropenylidene (a regular carbene), and imidazol-2-ylidene (N-heterocyclic carbene, NHC). The physical nature of these carbene⋯MX${}_{2}$ (X = H, Br) interactions has been described for the first time by the joint use of topological QTAIM-based IQA decomposition scheme, molecular orbital-based ETS-NOCV charge and energy decomposition method, as well as LED energy decomposition basing on the state-of-the-art DLPNO-CCSD(T) method.

All methods agree on the increasing bond strength in a series: Zn < Mg << Be. For beryllium and magnesium bonds, electrostatics proved to be the most important bond component (55–90% of total stabilization), followed by stabilization due to electron sharing (10–45% of total stabilization), while dispersion interactions proved to be marginal (2–5%). QTAIM/IQA underestimates electrostatic contribution for zinc bonds with respect to both ETS-NOCV and LED schemes which, similarly to Be and Mg species, identify the following importance of Zn–carbene bond constituents: $E_{\mathrm{elstat}}$ > $E_{\mathrm{orb}}$ > $E_{\mathrm{dispersion}}$. Both ETS-NOCV and LED also provide a consistent picture of these carbene bonds on an orbital level, with the σ carbene→MX${}_{2}$ donation strongly dominating over the MX${}_{2}\to$ carbene back-donation of π symmetry. Substitution of hydrogen atoms by bromines (X in MX${}_{2}$) strengthens the metal–carbene bond in all cases due to the amplification of electrostatic forces as well as donation and back-donation effects. The former is likely due to making metal atoms more electrophilic. Finally, the conformational preferences (planar vs twisted) are controlled by the nature of C–Zn bonding as unveiled by ETS-NOCV analyses performed along rotation coordinate pathways.

## Figures and Tables

**Table 1:** Some fundamental data characterizing cyclopropenylidene⋯MX${}_{2}$ (X = H, Br) and imidazol-2-ylidene⋯MBr${}_{2}$ dimers (M = Be, Mg, Zn): C⋯M and M-X distance, change of the MX bond length (in Å), XMX, CMX, LCL angles (in degrees), dissociation energy (in kcal/mol), charge transfer (in a.u.).

MX${}_{2}$	$d_{C\cdots M}$	$d_{\mathrm{MX}}$	${\Delta d}_{\mathrm{MX}}$	$\alpha_{\mathrm{XMX}}$	$\alpha_{\mathrm{CMX}}$	$\alpha_{\mathrm{LCL}}$	${\Delta \alpha }_{\mathrm{LCL}}$	$D_{0}$	CT ^*a*^
cyclopropenylidene									
BeH${}_{2}$	1.743	1.374	0.039	135.1	112.4	56.6	0.8	29.1	−0.317
MgH${}_{2}$	2.268	1.740	0.035	148.3	105.9	56.7	0.9	20.9	−0.273
ZnH${}_{2}$	2.121	1.575	0.036	144.6	107.7	57.3	0.9	15.2	−0.279
BeBr${}_{2}$	1.764	2.043	0.093	134.5	112.8	57.3	1.5	35.4	−0.414
MgBr${}_{2}$	2.206	2.376	0.052	145.4	107.3	57.0	1.3	32.1	−0.331
ZnBr${}_{2}$	2.061	2.298	0.083	137.1	111.5	57.3	1.5	28.2	−0.380
imidazol-2-ylidene									
BeBr${}_{2}$	1.765	2.059	0.109	132.9	113.6	104.2	3.5	48.6	−0.464
MgBr${}_{2}$	2.173	2.393 ^*b*^	0.069 ^*c*^	147.9	106.1 ^*d*^	103.5	2.8	43.7	−0.363
ZnBr${}_{2}$	2.037	2.311	0.097	139.2	110.4	104.2	3.4	41.2	−0.432

^*a*^ The charge transfer was calculated based on Hirshfeld’s atomic charges [126,127]. ^*b*^ The average value determined from the two values 2.386 Å and 2.400 Å. ^*c*^ The average value determined from the two values 0.062 Å and 0.076 Å. ^*d*^ The average value determined from the two values 102.9${}^{\circ }$ and 109.2${}^{\circ }$.

**Table 2:** The IQA-based energy (in a.u.) terms (see Methods) computed for the C⋯M interaction in the carbene⋯MX${}_{2}$ dimers.

MX${}_{2}$	$E_{\mathrm{neen}}$	$E_{\mathrm{nn}}$	$E_{\mathrm{ee}}$	$E_{\mathrm{ee},C}$	$E_{\mathrm{ee},\mathrm{xc}}$	${\%E}_{\mathrm{ee},\mathrm{xc}}$	$E_{\mathrm{elst}}$	$E_{\mathrm{int}}$
cyclopropenylidene								
BeH${}_{2}$	−12.2839	7.2846	4.6505	4.6963	−0.0458	13.1	−0.3030	−0.3488
MgH${}_{2}$	−32.4999	16.8017	15.5098	15.5405	−0.0307	16.3	−0.1577	−0.1884
ZnH${}_{2}$	−90.1277	44.8987	45.0634	45.1726	−0.1091	65.9	−0.0564	−0.1655
BeBr${}_{2}$	−12.1017	7.1990	4.5383	4.5810	−0.0428	11.7	−0.3217	−0.3644
MgBr${}_{2}$	−33.3934	17.2713	15.9040	15.9383	−0.0343	15.7	−0.1838	−0.2181
ZnBr${}_{2}$	−92.5816	46.2217	46.1682	46.2894	−0.1212	63.2	−0.0705	−0.1917
imidazol-2-ylidene								
BeBr${}_{2}$	−11.3609	7.1976	4.1093	4.1554	−0.0461	85.5	−0.0079	−0.0539
MgBr${}_{2}$	−32.0341	17.5351	14.5370	14.5767	−0.0397	−104.7	0.0777	0.0379
ZnBr${}_{2}$	−88.3086	46.7541	41.5418	41.6735	−0.1317	1037.0	0.1190	−0.0127

**Table 3:** Sums of the IQA-based energy terms (in kcal/mol) computed (MP2/6-311+G(d,p)) for groups of atoms reflecting the ETS-NOCV fragmentation, that is {carbene}⋯{MX${}_{2}\}$.

MX${}_{2}$	$E_{\mathrm{int}}$	$E_{\mathrm{elst}}$	${\%E}_{\mathrm{elst}}$	$E_{\mathrm{ee},\mathrm{xc}}$	${\%E}_{\mathrm{ee},\mathrm{xc}}$
cyclopropenylidene					
BeH${}_{2}$	−143.28	−73.68	51.4	−69.60	48.6
MgH${}_{2}$	−71.81	−38.55	53.7	−33.25	46.3
ZnH${}_{2}$	−91.45	−14.86	16.2	−76.59	83.8
BeBr${}_{2}$	−162.74	−90.98	55.9	−71.76	44.1
MgBr${}_{2}$	−88.25	−49.41	56.0	−38.85	44.0
ZnBr${}_{2}$	−105.00	−18.61	17.7	−86.38	82.3
imidazol-2-ylidene					
BeBr${}_{2}$	−190.24	−110.00	57.8	−80.27	42.2
MgBr${}_{2}$	−113.04	−63.87	56.5	−49.17	43.5
ZnBr${}_{2}$	−127.25	−26.19	20.6	−101.05	79.4

**Table 4:** The ETS-NOCV-based energy (in kcal/mol) contributions to carbene⋯MX${}_{2}$ bonding.

MX${}_{2}$	${\Delta E}_{\mathrm{int}}$	${\Delta E}_{\mathrm{elst}}$	${\Delta E}_{\mathrm{Pauli}}$	${\Delta E}_{\mathrm{disp}}$	${\Delta E}_{\mathrm{orb}}$	${\Delta E}_{\mathrm{orb}}^{\sigma }$	${\Delta E}_{\mathrm{orb}}^{\pi }$	${\Delta E}_{\mathrm{orb}}^{\mathrm{NH}\cdots \mathrm{Br}}$
cyclopropenylidene								
BeH${}_{2}$	−45.69	−69.29 (56%)	77.03	−2.56 (2%)	−50.87 (41%)	−40.33	−10.11	n/a
MgH${}_{2}$	−26.20	−45.85 (70%)	39.55	−2.58 (4%)	−17.32 (26%)	−14.16	−2.88	n/a
ZnH${}_{2}$	−23.63	−75.38 (68%)	87.86	−2.87 (3%)	−33.24 (30%)	−27.31	−5.19	n/a
BeBr${}_{2}$	−52.23	−82.67 (58%)	90.98	−4.90 (3%)	−55.64 (39%)	−47.43	−6.34	n/a
MgBr${}_{2}$	−38.57	−57.23 (69%)	44.25	−4.14 (5%)	−21.44 (26%)	−17.67	−2.56	n/a
ZnBr${}_{2}$	−37.74	−95.45 (67%)	104.58	−4.52 (3%)	−42.34 (30%)	−36.96	−3.53	n/a
imidazol-2-ylidene								
BeBr${}_{2}$	−66.86	−103.25 (61%)	102.10	−6.61 (4%)	−59.10 (35%)	−47.92	−5.24	−1.71
MgBr${}_{2}$	−50.49	−77.00 (71%)	58.24	−6.01 (5%)	−25.72 (24%)	−18.57	−3.13	−1.10
ZnBr${}_{2}$	−50.92	−122.82 (68%)	128.22	−6.45 (4%)	−49.87 (28%)	−42.15	−3.70	−1.11

**Table 5:** DPLNO-CCSD(T)/ccpv-tz Local Energy Decomposition analysis of the {carbene}⋯{MX${}_{2}$} bonding (carbene names shortened for clarity: cpy = cyclopropenylidene and imi = imidazol-2-ylidene). Energies in kcal/mol.

	cpy–BeBr${}_{2}$	cpy–MgBr${}_{2}$	cpy–ZnBr${}_{2}$	imi–BeBr${}_{2}$	imi–MgBr${}_{2}$	imi–ZnBr${}_{2}$
E${}_{\mathrm{int}}$	−39.60	−34.28	−29.08	−54.28	−46.37	−42.63
E${}_{\mathrm{elst}}^{\mathrm{ref}}$	−218.44	−152.51	−327.12	−251.84	−190.31	−393.52
E${}_{\mathrm{exch}}^{\mathrm{ref}}$	−20.12	−11.84	−33.73	−20.84	14.59	−39.32
E${}_{\mathrm{geo}-\mathrm{prep}}^{\mathrm{ref}}$	14.88	6.23	10.76	17.58	6.73	11.24
E${}_{\mathrm{el}-\mathrm{prep}}^{\mathrm{ref}}$	190.33	126.75	325.62	210.63	158.28	387.67
E${}_{\mathrm{non}-\mathrm{disp}}^{C}$	-0.69	0.12	1.91	−2.74	−1.83	−0.17
E${}_{\mathrm{disp}}^{C}$	−5.35	−2.93	−6.14	−6.43	−4.10	−7.67
E${}_{T}^{C}$	−0.21	−0.10	−0.38	−0.63	−0.55	−0.85
CT 1→2	−16.96	−9.52	−13.44	−19.17	−11.11	−16.32
CT 2→1	−3.33	−2.43	−7.58	−3.56	−2.53	−8.14

## Data Availability

Data available from the authors on reasonable request.

## Supplementary Materials

The following are available at https://www.mdpi.com/article/10.3390/ijms232314668/s1.

## Abbreviations

The following abbreviations are used in this manuscript:

QTAIM	Quantum Theory of Atoms in Molecules
IQA	the Interacting Quantum Atoms approach
ETS	the Extended Transition State method
NOCV	the Natural Orbitals for Chemical Valence method
DLPNO	Domain-based Localized Pair-Natural Orbital
CCSD(T)	the coupled cluster (CC) single-double-triple method
LED	the Local Energy Decomposition scheme

## References

[1] Kirmse W. (1964). Carbene Chemistry.

[2] Hubert A.J. (1983). Catalysis in C_1_ Chemistry.

[3] Schubert U. (1989). Advances in Metal Carbene Chemistry.

[4] Herrmann W.A., Köcher C. (1997). N-Heterocyclic Carbenes. Angew. Chem. Int. Ed. Engl..

[5] Bourissou D., Guerret O., Gabbaï F.P., Bertrand G. (2000). Stable Carbenes. Chem. Rev..

[6] Bertrande G. (2002). Carbene Chemistry: From Fleeting Intermediates to Powerful Reagents.

[7] Moss R.A., Platz M.S., Jones M. (2005). Reactive Intermediate Chemistry.

[8] Scott N.M., Nolan S.P. (2005). Stabilization of Organometallic Species Achieved by the Use of N-Heterocyclic Carbene (NHC) Ligands. Eur. J. Inorg. Chem..

[9] Nolan S.P. (2006). N-Heterocyclic Carbenes in Synthesis.

[10] Carey F.A., Sundberg R.J. (2007). Carbenes, Part B: Reactions and Synthesis. Advanced Organic Chemistry.

[11] Díez-González S., Nolan S.P. (2007). Stereoelectronic parameters associated with N-heterocyclic carbene (NHC) ligands: A quest for understanding. Coord. Chem. Rev..

[12] Jacobsen H., Correa A., Poater A., Costabile C., Cavallo L. (2009). Understanding the M–(NHC) (NHC = N-heterocyclic carbene) bond. Coord. Chem. Rev..

[13] de Frémont P., Marion N., Nolan S.P. (2009). Carbenes: Synthesis, properties, and organometallic chemistry. Coord. Chem. Rev..

[14] Moss R.A., Doyle M.P. (2013). Contemporary Carbene Chemistry.

[15] Nelson D.J., Nolan S.P. (2013). Quantifying and understanding the electronic properties of N-heterocyclic carbenes. Chem. Soc. Rev..

[16] Bellemin-Laponnaz S., Dagorne S. (2014). Group 1 and 2 and Early Transition Metal Complexes Bearing N-Heterocyclic Carbene Ligands: Coordination Chemistry, Reactivity, and Applications. Chem. Rev..

[17] Hopkinson M.N., Richter C., Schedler M., Glorius F. (2014). An overview of N-heterocyclic carbenes. Nature.

[18] Nesterov V., Reiter D., Bag P., Frisch P., Holzner R., Porzelt A., Inoue S. (2018). NHCs in Main Group Chemistry. Chem. Rev..

[19] Pople J.A., Raghavachari K., Frisch M.J., Binkley J.S., Schleyer P.v.R. (1983). Comprehensive Theoretical Study of Isomers and Rearrangement Barriers of Even Electron Polyatomic Molecules H_m_ABH_n_ (A, B = C, N, O, and F). J. Am. Chem. Soc..

[20] Pople J.A. (1986). A theoretical search for the methylenefluoronium ylide. Chem. Phys. Lett..

[21] Arduengo A.J., Gamper S.F., Tamm M., Calabrese J.C., Davidson F., Craig H.A. (1995). A Bis(carbene)–Proton Complex: Structure of a C–H–C Hydrogen Bond. J. Am. Chem. Soc..

[22] Alkorta I., Elguero J. (1996). Carbenes and Silylenes as Hydrogen Bond Acceptors. J. Phys. Chem..

[23] Jabłoński M., Palusiak M. (2009). Divalent carbon atom as the proton acceptor in hydrogen bonding. Phys. Chem. Chem. Phys..

[24] Giffin N.A., Makramalla M., Hendsbee A.D., Robertson K.N., Sherren C., Pye C.C., Masuda J.D., Clyburne J.A.C. (2011). Anhydrous TEMPO-H: Reactions of a good hydrogen atom donor with low-valent carbon centres. Org. Biomol. Chem..

[25] Gerbig D., Ley D. (2013). Computational methods for contemporary carbene chemistry. WIREs Comput. Mol. Sci..

[26] Samanta R.C., De Sarkar S., Fröhlich R., Grimme S., Studer A. (2013). N-Heterocyclic carbene (NHC) catalyzed chemoselective acylation of alcohols in the presence of amines with various acylating reagents. Chem. Sci..

[27] Jabłoński M. (2022). Coexistence of the Carbene⋯H-D Hydrogen Bond and Other Accompanying Interactions in Forty Dimers of N-Heterocyclic-Carbenes (I, IMe_2_, I^i^Pr_2_, I^t^Bu_2_, IMes_2_, IDipp_2_, IAd_2_; I = imidazol-2-ylidene) and Some Fundamental Proton Donors (HF, HCN, H_2_O, MeOH, NH_3_). Molecules.

[28] Li Q., Wang H., Liu Z., Li W., Cheng J., Gong B., Sun J. (2009). Ab Initio Study of Lithium-Bonded Complexes with Carbene as an Electron Donor. J. Phys. Chem. A.

[29] Wang Y., Xie Y., Abraham M.Y., Wei P., Schaefer H.F., Schleyer P.v.R., Robinson G.H. (2010). A Viable Anionic N-Heterocyclic Dicarbene. J. Am. Chem. Soc..

[30] Zhi-Feng L., Sheng Y., Hui-Xue L. (2010). Theoretical prediction characters of unconventional weak bond with carbene as electron donors and Li–Y (Y = OH, H, F, NC and CN) as electron acceptors. J. Mol. Struct. THEOCHEM.

[31] Herrmann W.A., Runte O., Artus G. (1995). Synthesis and structure of an ionic beryllium—“Carbene” complex. J. Organomet. Chem..

[32] Gilliard R.J., Abraham M.Y., Wang Y., Wei P., Xie Y., Quillian B., Schaefer H.F., Schleyer P.v.R., Robinson G.H. (2012). Carbene-Stabilized Beryllium Borohydride. J. Am. Chem. Soc..

[33] Arrowsmith M., Hill M.S., Kociok-Köhn G., MacDougall D.J., Mahon M.F. (2012). Beryllium-Induced C–N Bond Activation and Ring Opening of an N-Heterocyclic Carbene. Angew. Chem. Int. Ed..

[34] Walley J.E., Wong Y.-O., Freeman L.A., Dickie D.A., Gilliard R.J. (2019). N-Heterocyclic Carbene-Supported Aryl- and Alk- oxides of Beryllium and Magnesium. Catalysts.

[35] Jabłoński M. (2021). Study of Beryllium, Magnesium, and Spodium Bonds to Carbenes and Carbodiphosphoranes. Molecules.

[36] Arduengo A.J., Dias H.V.R., Davidson F., Harlow R.L. (1993). Carbene adducts of magnesium and zinc. J. Organomet. Chem..

[37] Arrowsmith M., Hill M.S., MacDougall D.J., Mahon M.F. (2009). A Hydride-Rich Magnesium Cluster. Angew. Chem. Int. Ed..

[38] Arnold P.L., Casely I.J., Turner Z.R., Bellabarba R., Tooze R.B. (2009). Magnesium and zinc complexes of functionalised, saturated N-heterocyclic carbene ligands: Carbene lability and functionalisation, and lactide polymerisation catalysis. Dalton Trans..

[39] Arduengo A.J., Dias H.V.R., Calabrese J.C., Davidson F. (1992). A Stable Carbene-Alane Adduct. J. Am. Chem. Soc..

[40] Li X.-W., Su J., Robinson G.H. (1996). Syntheses and molecular structure of organo-group 13 metal carbene complexes. Chem. Commun..

[41] Hibbs D.E., Hursthouse M.B., Jones C., Smithies N.A. (1998). Synthesis, crystal and molecular structure of the first indium trihydride complex, [InH_3_{CN(Pr^i^)C_2_Me_2_N(Pr^i^)}]. Chem. Commun..

[42] Merceron N., Miqueu K., Baceiredo A., Bertrand G. (2002). Stable (Amino)(phosphino)carbenes: Difunctional Molecules. J. Am. Chem. Soc..

[43] Wang Y., Robinson G.H. (2009). Unique homonuclear multiple bonding in main group compounds. Chem. Commun..

[44] Del Bene J.E., Alkorta I., Elguero J. (2017). Carbon–Carbon Bonding between Nitrogen Heterocyclic Carbenes and CO_2_. J. Phys. Chem. A.

[45] Liu M., Li Q., Li W., Cheng J. (2017). Carbene tetrel-bonded complexes. Struct. Chem..

[46] Wang Y., Xie Y., Abraham M.Y., Gilliard R.J., Wei P., Schaefer H.F., Schleyer P.v.R., Robinson G.H. (2010). Carbene-Stabilized Parent Phosphinidene. Organometallics.

[47] Abraham M.Y., Wang Y., Xie Y., Wei P., Schaefer H.F., Schleyer P.v.R., Robinson G.H. (2010). Carbene Stabilization of Diarsenic: From Hypervalency to Allotropy. Chem. Eur. J..

[48] Patel D.S., Bharatam P.V. (2011). Divalent N(I) Compounds with Two Lone Pairs on Nitrogen. J. Phys. Chem. A.

[49] Zhao Q., Feng D., Sun Y., Hao J., Cai Z. (2011). Theoretical Investigations on the Weak Nonbonded C=S⋯CH_2_ Interactions: Chalcogen-Bonded Complexes With Singlet Carbene as an Electron Donor. Int. J. Quant. Chem..

[50] Arduengo A.J., Kline M., Calabrese J.C., Davidson F. (1991). Synthesis of a Reverse Ylide from a Nucleophilic Carbene. J. Am. Chem. Soc..

[51] Kuhn N., Kratz T., Henkel G. (1993). A Stable Carbene Iodine Adduct: Secondary Bonding in 1,3-Diethyl-2-iodo-4,5-dimethylimidazolium Iodide. J. Chem. Soc. Chem. Commun..

[52] Li Q., Wang Y., Liu Z., Li W., Cheng J., Gong B., Sun J. (2009). An unconventional halogen bond with carbene as an electron donor: An ab initio study. Chem. Phys. Lett..

[53] Esrafili M.D., Mohammadirad N. (2013). Insights into the strength and nature of carbene⋯halogen bond interactions: A theoretical perspective. J. Mol. Model..

[54] Esrafili M.D., Sabouri A. (2017). Carbene–aerogen bonds: An Ab Initio Study. Mol. Phys..

[55] Cases M., Frenking G., Duran M., Solà M. (2002). Molecular Structure and Bond Characterization of the Fischer-Type Chromium–Carbene Complexes (CO)_5_Cr=C(X)R (X = H, OH, OCH_3_, NH_2_, NHCH_3_ and R = H, CH_3_, CH=CH_2_, Ph, C≡CH). Organometallics.

[56] Nemcsok D., Wichmann K., Frenking G. (2004). The Significance of *π* Interactions in Group 11 Complexes with N-Heterocyclic Carbenes. Organometallics.

[57] Frenking G., Solà M., Vyboishchikov S.F. (2005). Chemical bonding in transition metal carbene complexes. J. Organomet. Chem..

[58] Tonner R., Heydenrych G., Frenking G. (2007). Bonding Analysis of N-Heterocyclic Carbene Tautomers and Phosphine Ligands in Transition-Metal Complexes: A Theoretical Study. Chem. Asian J..

[59] Radius U., Bickelhaupt F.M. (2008). Bonding of Imidazol-2-ylidene Ligands in Nickel Complexes. Organometallics.

[60] Srebro M., Michalak A. (2009). Theoretical Analysis of Bonding in N-Heterocyclic Carbene–Rhodium Complexes. Inorg. Chem..

[61] Andrada D.M., Holzmann N., Hamadi T., Frenking G., Beilstein J. (2015). Direct estimate of the internal *π*-donation to the carbene centre within N-heterocyclic carbenes and related molecules. Org. Chem..

[62] Santoro O., Nahra F., Cordes D.B., Slawin A.M.Z., Nolan S.P., Cazin C.S.J. (2016). Synthesis, characterization and catalytic activity of stable [(NHC)H][ZnXY_2_] (NHC = *N*-Heterocyclic carbene, X, Y = Cl, Br) species. J. Mol. Catal..

[63] Dagorne S. (2018). Recent Developments on N-Heterocyclic Carbene Supported Zinc Complexes: Synthesis and Use in Catalysis. Synthesis.

[64] Procter R.J., Uzelac M., Cid J., Rushworth P.J., Ingleson M.J. (2019). Low-Coordinate NHC–Zinc Hydride Complexes Catalyze Alkyne C–H Borylation and Hydroboration Using Pinacolborane. ACS Catal..

[65] Specklin D., Fliedel C., Dagorne S. (2021). Recent Representative Advances on the Synthesis and Reactivity of N-Heterocyclic-Carbene-Supported Zinc Complexes. Chem. Rec..

[66] Arduengo A.J., Davidson F., Krafczyk R., Marshall W.J., Tamm M. (1998). Adducts of Carbenes with Group II and XII Metallocenes. Organometallics.

[67] Abernethy C.D., Baker R.J., Cole M.L., Davies A.J., Jones C. (2003). Reactions of a carbene stabilised indium trihydride complex, [InH_3_{CN(Mes)-C_2_H_2_N(Mes)}] Mes = mesityl, with transition metal complexes. Trans. Met. Chem..

[68] Wang D., Wurst K., Buchmeiser M.R. (2004). N-heterocyclic carbene complexes of Zn(II): Synthesis, X-ray structures and reactivity. J. Organomet. Chem..

[69] Jensen T.R., Breyfogle L.E., Hillmyer M.A., Tolman W.B. (2004). Stereoelective polymerization of D,L-lactide using N-heterocyclic carbene based compounds. Chem. Commun..

[70] Jensen T.R., Schaller C.P., Hillmyer M.A., Tolman W.B. (2005). Zinc N-heterocyclic carbene complexes and their polymerization of D,L-lactide. J. Organomet. Chem..

[71] Anantharaman G., Elango K. (2007). Synthesis and Characterization of NHC-Stabilized Zinc Aryloxide and Zinc Hydroxyaryloxide. Organometallics.

[72] Budagumpi S., Endud S. (2013). Group XII Metal–N-Heterocyclic Carbene Complexes: Synthesis, Structural Diversity, Intramolecular Interactions, and Applications. Organometallics.

[73] Schnee G., Fliedel C., Avilés T., Dagorne S. (2013). Neutral and Cationic N-Heterocyclic Carbene Zinc Adducts and the BnOH/Zn(C_6_F_5_)_2_ Binary Mixture–Characterization and Use in the Ring-Opening Polymerization of *β*-Butyrolactone, Lactide, and Trimethylene Carbonate. Eur. J. Inorg. Chem..

[74] Fliedel C., Vila-Viçosa D., Calhorda M.J., Dagorne S., Avilés T. (2014). Dinuclear Zinc–N-Heterocyclic Carbene Complexes for Either the Controlled Ring-Opening Polymerization of Lactide or the Controlled Degradation of Polylactide Under Mild Conditions. ChemCatChem.

[75] Collins L.R., Moffat L.A., Mahon M.F., Jones M.D., Whittlesey M.K. (2016). Lactide polymerisation by ring-expanded NHC complexes of zinc. Polyhedron.

[76] Tian J., Chen Y., Vayer M., Djurovic A., Guillot R., Guermazi R., Dagorne S., Bour C., Gandon V. (2020). Exploring the Limits of *π*-Acid Catalysis Using Strongly Electrophilic Main Group Metal Complexes: The Case of Zinc and Aluminium. Chem. Eur. J..

[77] Roy M.M.D., Baird S.R., Ferguson M.J., Rivard E. (2021). Toward N-heterocyclic carbene stabilized zinc sulfides. Mendeleev Commun..

[78] Jabłoński M. (2021). Theoretical Study of N-Heterocyclic-Carbene–ZnX_2_ (X = H, Me, Et) Complexes. Materials.

[79] Schoeller W.W. (1980). Electrophilicity and nucleophilicity in singlet carbenes. II. Electrophilic selectivity. Tetrahedron Lett..

[80] Goumri-Magnet S., Polishchuk O., Gornitzka H., Marsden C.J., Baceiredo A., Bertrand G. (1999). The Electrophilic Behavior of Stable Phosphanylcarbenes Towards Phosphorus Lone Pairs. Angew. Chem. Int. Ed..

[81] Moss R.A., Wang L., Cang H., Krogh-Jespersen K. (2017). Extremely reactive carbenes: Electrophiles and nucleophiles. J. Phys. Org. Chem..

[82] Jabłoński M. (2018). The first theoretical proof of the existence of a hydride-carbene bond. Chem. Phys. Lett..

[83] Jabłoński M. (2019). In search for a hydride-carbene bond. J. Phys. Org. Chem..

[84] Yourdkhani S., Jabłoński M. (2019). Physical nature of silane⋯carbene dimers revealed by state-of-the-art ab initio calculations. J. Comput. Chem..

[85] Yáñez M., Sanz P., Mó O., Alkorta I., Elguero J. (2009). Beryllium Bonds, Do They Exists?. J. Chem. Theory Comput..

[86] Martín-Sómer A., Lamsabhi A.M., Mó O., Yáñez M. (2012). The importance of deformation on the strength of beryllium bonds. Comput. Theor. Chem..

[87] Eskandari K. (2012). Characteristics of beryllium bonds: A QTAIM study. J. Mol. Model..

[88] Villanueva E.F., Mó O., Yáñez M. (2014). On the existence and characteristics of *π*-beryllium bonds. Phys. Chem. Chem. Phys..

[89] Zhong A., Chen D., Li R. (2015). Revisiting the beryllium bonding interactions from energetic and wavefunction perspectives. Chem. Phys. Lett..

[90] Eskandari K. (2016). Nature of beryllium bonds in view of interacting quantum atoms and natural energy decomposition analysis. Comput. Theor. Chem..

[91] Montero-Campillo M.M., Mó O., Yáñez M., Alkorta I., Elguero J. (2019). The beryllium bond. Adv. Inorg. Chem..

[92] Alkorta I., Legon A.C. (2019). Non-Covalent Interactions Involving Alkaline-Earth Atoms and Lewis Bases B: An ab Initio Investigation of Beryllium and Magnesium Bonds, B⋯MR_2_ (M = Be or Mg, and R = H, F or CH_3_). Inorganics.

[93] Jabłoński M. (2019). On the Uselessness of Bond Paths Linking Distant Atoms and on the Violation of the Concept of Privileged Exchange Channels. Chem. Open.

[94] Yang X., Li Q., Cheng J., Li W. (2013). A new interaction mechanism of LiNH_2_ with MgH_2_: Magnesium bond. J. Mol. Model..

[95] Xu H.-L., Li Q.-Z., Scheiner S. (2018). Effect of magnesium bond on the competition between hydrogen and halogen bonds and the induction of proton and halogen transfer. ChemPhysChem.

[96] Sanz P., Montero-Campillo M.M., Mó O., Yáñez M., Alkorta I., Elguero J. (2018). Intramolecular magnesium bonds in malonaldehyde-like systems: A critical view of the resonance-assisted phenomena. Theor. Chem. Acc..

[97] Lupinetti A.J., Jonas V., Thiel W., Strauss S.H., Frenking G. (1999). Trends in Molecular Geometries and Bond Strengths of the Homoleptic d^10^ Metal Carbonyl Cations [M(CO)_n_]^x+^ (M^x+^ = Cu^+^, Ag^+^, Au^+^, Zn^2+^, Cd^2+^, Hg^2+^; *n* = 1–6): A Theoretical Study. Chem. Eur. J..

[98] Joy J., Jemmis E.D. (2017). Contrasting Behavior of the Z Bonds in X–Z⋯Y Weak Interactions: Z = Main Group Elements Versus the Transition Metals. Inorg. Chem..

[99] Wang S.R., Arrowsmith M., Braunschweig H., Dewhurst R.D., Dömling M., Mattock J.D., Pranckevicius C., Vargas A. (2017). Monomeric 16-Electron *π*-Diborene Complexes of Zn(II) and Cd(II). J. Am. Chem. Soc..

[100] Kalhor P., Wang Y., Yu Z. (2021). The Structures of ZnCl_2_-Ethanol Mixtures, a Spectroscopic and Quantum Chemical Calculation Study. Molecules.

[101] Bader R.F.W. (1990). Atoms in Molecules: A Quantum Theory.

[102] Cremer D., Kraka E. (1984). Chemical Bonds without Bonding Electron Density–Does the Difference Electron-Density Analysis Suffice for a Description of the Chemical Bond?. Angew. Chem. Int. Ed. Engl..

[103] Bader R.F.W., Stephens M.E. (1975). Spatial Localization of the Electronic Pair and Number Distributions in Molecules. J. Am. Chem. Soc..

[104] Fradera X., Austen M.A., Bader R.F.W. (1999). The Lewis Model and Beyond. J. Phys. Chem. A.

[105] Fradera X., Poater J., Simon S., Duran M., Solà M. (2002). Electron-pairing analysis from localization and delocalization indices in the framework of the atoms-in-molecules theory. Theor. Chem. Acc..

[106] Firme C.L., Antunes O.A.C., Esteves P.M. (2009). Relation between bond order and delocalization index of QTAIM. Chem. Phys. Lett..

[107] Rafat M., Popelier P.L.A., Matta C.F., Boyed R.J. (2007). The Quantum Theory of Atoms in Molecules: From Solid State to DNA and Drug Design.

[108] García-Revilla M., Francisco E., Popelier P.L.A., Pendás A.M. (2013). Domain-Averaged Exchange-Correlation Energies as a Physical Underpinning for Chemical Graphs. ChemPhysChem.

[109] Johnson E.R., Keinan S., Mori-Sánchez P., Contreras-García J., Cohen A.J., Yang W. (2010). Revealing Noncovalent Interactions. J. Am. Chem. Soc..

[110] Contreras-García J., Johnson E.R., Keinan S., Chaudret R., Piquemal J.-P., Beratan D.N., Yang W. (2011). NCIPLOT: A Program for Plotting Noncovalent Interaction Regions. J. Chem. Theory Comput..

[111] Blanco M.A., Pendás A.M., Francisco E. (2005). Interacting Quantum Atoms: A Correlated Energy Decomposition Scheme Based on the Quantum Theory of Atoms in Molecules. J. Chem. Theory Comput..

[112] Guevara-Vela J.M., Francisco E., Rocha-Rinza T., Pendás A.M. (2020). Interacting Quantum Atoms–A Review. Molecules.

[113] Ziegler T., Rauk A. (1977). On the calculation of bonding energies by the Hartree Fock Slater method. Theoret. Chim. Acta (Berl.).

[114] Mitoraj M., Michalak A. (2007). Donor–Acceptor Properties of Ligands from the Natural Orbitals for Chemical Valence. Organometallics.

[115] Mitoraj M., Michalak A. (2007). Natural orbitals for chemical valence as descriptors of chemical bonding in transition metal complexes. J. Mol. Model..

[116] Michalak A., Mitoraj M., Ziegler T. (2008). Bond Orbitals from Chemical Valence Theory. J. Phys. Chem. A.

[117] Mitoraj M.P., Michalak A., Ziegler T. (2009). A Combined Charge and Energy Decomposition Scheme for Bond Analysis. J. Chem. Theory Comput..

[118] Mitoraj M.P., Michalak A., Ziegler T. (2009). On the Nature of the Agostic Bond between Metal Centers and *β*-Hydrogen Atoms in Alkyl Complexes. An Analysis Based on the Extended Transition State Method and the Natural Orbitals for Chemical Valence Scheme (ETS-NOCV). Organometallics.

[119] Saebø S., Pulay P. (1993). Local Treatment of Electron Correlation. Annu. Rev. Phys. Chem.

[120] Neese F., Wennmohs F., Hansen A. (2009). Efficient and accurate local approximations to coupled-electron pair approaches: An attempt to revive the pair natural orbital method. J. Chem. Phys..

[121] Neese F., Hansen A., Liakos D.G. (2009). Efficient and accurate approximations to the local coupled cluster singles doubles method using a truncated pair natural orbital basis. J. Chem. Phys..

[122] Riplinger C., Sandhoefer B., Hansen A., Neese F. (2013). Natural triple excitations in local coupled cluster calculations. J. Chem. Phys..

[123] Guo Y., Riplinger C., Becker U., Liakos D.G., Minenkov Y., Cavallo L., Neese F. (2018). Communication: An improved linear scaling perturbative triples correction for the domain based local pair-natural orbital based singles and doubles coupled cluster method [DLPNO-CCSD(T)]. J. Chem. Phys..

[124] Altun A., Saitow M., Neese F., Bistoni G. (2019). Local Energy Decomposition of Open-Shell Molecular Systems in the Domain-Based Local Pair Natural Orbital Coupled Cluster Framework. J. Chem. Theory Comput..

[125] Altun A., Izsák R., Bistoni G. (2021). Local energy decomposition of coupled-cluster interaction energies: Interpretation, benchmarks, and comparison with symmetry-adapted perturbation theory. Int. J. Quantum Chem..

[126] Hirshfeld F.L. (1977). Bonded-Atom Fragments for Describing Molecular Charge Densities. Theor. Chim. Acta.

[127] Ritchie J.P., Bachrach S.M. (1987). Some Methods and Applications of Electron Density Distribution Analysis. J. Comput. Chem..

[128] Ritchie J.P. (1985). Electron Density Distribution Analysis for Nitromethane, Nitromethide, and Nitramide. J. Am. Chem. Soc..

[129] Wiberg K.B., Rablen P.R. (2018). Atomic Charges. J. Org. Chem..

[130] Jabłoński M., Krygowski T.M. (2020). Study of the influence of intermolecular interaction on classical and reverse substituent effects in para-substituted phenylboranes. New J. Chem..

[131] Walsh A.D. (1953). The Electronic Orbitals, Shapes, and Spectra of Polyatomic Molecules. Part I. AH_2_ Molecules. J. Chem. Soc..

[132] Chai J.-D., Head-Gordon M. (2008). Long-range corrected hybrid density functionals with damped atom–atom dispersion corrections. Phys. Chem. Chem. Phys..

[133] Hohenberg P., Kohn W. (1964). Inhomogeneous Electron Gas. Phys. Rev..

[134] Kohn W., Sham L.J. (1965). Self-Consistent Equations Including Exchange and Correlation Effects. Phys. Rev..

[135] Parr R.G., Yang W. (1989). Density-Functional Theory of Atoms and Molecules.

[136] Mardirossian N., Head-Gordon M. (2017). Thirty years of density functional theory in computational chemistry: An overview and extensive assessment of 200 density functionals. Mol. Phys..

[137] Krishnan R., Binkley J.S., Seeger R., Pople J.A. (1980). Self-consistent molecular orbital methods. XX. A basis set for correlated wave functions. J. Chem. Phys..

[138] McLean A.D., Chandler G.S. (1980). Contracted Gaussian basis sets for molecular calculations. I. second row atoms, Z = 11–18. J. Chem. Phys..

[139] Clark T., Chandrasekhar J., Spitznagel G.W., Schleyer P.v.R. (1983). Efficient Diffuse Function-Augmented Basis Sets for Anion Calculations. III. The 3-21+G Basis Set for First-Row Elements, Li–F. J. Comput. Chem..

[140] Frisch M.J., Pople J.A., Binkley J.S. (1984). Self-consistent molecular orbital methods 25. Supplementary functions for Gaussian basis sets. J. Chem. Phys..

[141] Curtiss L.A., McGrath M.P., Blandeau J.-P., Davis N.E., Binning R.C., Radom L. (1995). Extension of Gaussian-2 theory to molecules containing third-row atoms Ga–Kr. J. Chem. Phys..

[142] Jensen F. (2007). Introduction to Computational Chemistry.

[143] Pritchard B.P., Altarawy D., Didier B., Gibson T.D., Windus T.L. (2019). New Basis Set Exchange: An Open, Up-to-Date Resource for the Molecular Sciences Community. J. Chem. Inf. Model..

[144] Frisch M.J., Trucks G.W., Schlegel H.B., Scuseria G.E., Robb M.A., Cheeseman J.R., Scalmani G., Barone V., Mennucci B., Petersson G.A. (2013). Gaussian 09.

[145] Keith T.A. (2015). AIMAll (Version 15.05.18).

[146] Møller C., Plesset M.S. (1934). Note on an Approximation Treatment for Many-Electron Systems. Phys. Rev..

[147] Head-Gordon M., Pople J.A., Frisch M.J. (1988). MP2 Energy Evaluation by Direct Methods. Chem. Phys. Lett..

[148] Becke A.D. (1988). Density-fnnctional exchange-energy approximation with correct asymptotic behavior. Phys. Rev. A.

[149] Lee C., Yang W., Parr R.G. (1988). Development of the Colle-Salvetti correlation-energy formula into a functional of the electron density. Phys. Rev. B.

[150] Grimme S., Ehrlich S., Goerigk L. (2011). Effect of the Damping Function in Dispersion Corrected Density Functional Theory. J. Comput. Chem..

[151] van Lenthe E., Baerends E.J., Snijder J.G. (1993). Relativistic regular two-component Hamiltonians. J. Chem. Phys..

[152] Snijder J.G., Sadlej A.J. (1996). Perturbation versus variation treatment of regular relativistic Hamiltonians. Chem. Phys. Lett..

[153] Baerends E.J., Ziegler T., Atkins A.J., Autschbach J., Baseggio O., Bashford D., Bééces A., Bickelhaupt F.M., Bo C., Boerrigter P.M. (2022). ADF 2022.1.

[154] Sagan F., Mitoraj M.P., Broclawik E., Borowski T., Radoń M. (2019). Non-covalent Interactions in Selected Transition Metal Complexes. Transition Metals in Coordination Environments: Computational Chemistry and Catalysis Viewpoints.

[155] Stasyuk O.A., Sedlak R., Fonseca-Guerra C., Hobza P. (2018). Comparison of the DFT-SAPT and Canonical EDA Schemes for the Energy Decomposition of Various Types of Noncovalent Interactions. J. Chem. Theory Comput..

