# Supplementary material for: Nature of Beryllium, Magnesium, and Zinc Bonds in Carbene⋯MX2 (M = Be, Mg, Zn; X = H, Br) Dimers Revealed by the IQA, ETS-NOCV and LED Methods
Source: Int J Mol Sci. 2022 Nov 24;23(23):14668. doi: 10.3390/ijms232314668 (PMC9738500; doi:10.3390/ijms232314668)
Supplement: Supplementary file 1 [file ijms-23-14668-s001.zip › ijms-2019654-supplementary.pdf]

Supplementary Materials: Nature of Beryllium, Magnesium, and Zinc Bonds in Carbene$\cdots MX_2$ (M = Be, Mg, Zn; X = H, Br) Dimers Revealed by the IQA, ETS-NOCV and LED Methods

Filip Sagan [1], Mariusz Mitoraj [1] and Mirosław Jabłoński [2,*]

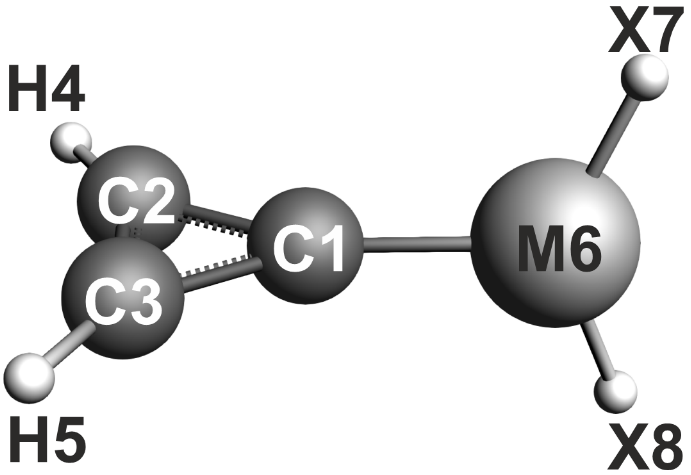

Figure S1. Model MX_2-cyclopropenylidene molecule with atom numbering; M = (Be, Mg, Zn), X = (H,Br).

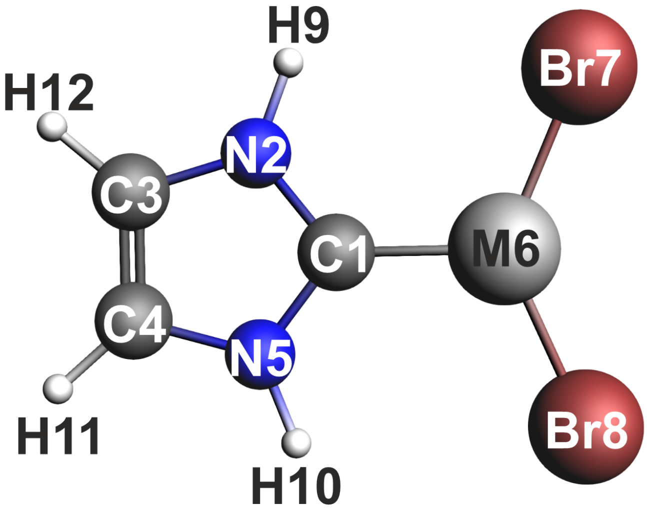

Figure S2. Model MBr_2-imidazol-2-ylidene molecule with atom numbering; M = (Be, Mg, Zn).

Table S1. Dissociation energies (based on the geometries optimized in ωB97X-D/6-311++G**) of the systems under study calculated in all methods used herein: ωB97X-D/6-311++G**, BLYP-D3(BJ)/TZP, MP2/6-311+G*, DLPNO-CCSD(T)/cc-pVTZ. All energies in kcal/mol.

MX_2	ωB97X-D	BLYP-D3(BJ)	MP2	DLPNO-CCSD(T)
cyclopropenylidene				
BeH_2	29.1	34.4	28.4	23.9
MgH_2	20.9	22.2	20.4	23.4
ZnH_2	15.2	14.9	14.7	15.5
$BeBr_2$	35.4	39.3	37.6	39.6
$MgBr_2$	32.1	33.8	32.5	34.3
$ZnBr_2$	28.2	28.5	29.8	29.1
imidazol-2-ylidene				
$BeBr_2$	48.6	51.3	52.7	54.3
$MgBr_2$	43.7	45.2	45.5	46.4
$ZnBr_2$	41.2	41.2	44.7	42.6

Table S2. The most stabilizing IQA atomic pair energies (in kcal/mol) for imidazol-2-ylidene complexes with MBr_2, computed at the MP2/6-311+G(d,p) level of theory. Atom numbering as presented on Fig. S2

Atom A	Atom B	E_{int}^{AB}	E_{elst}^{AB}	$E_{ee,XC}^{AB}$
BeBr2–imidazol-2-ylidene				
Be6	N2	-253.35	-253.83	-0.52
Be6	N5	-253.36	-252.84	-0.52
Br7	H9	-48.08	-45.48	-2.60
Br8	H10	-48.08	-45.48	-2.60
Br7	C1	-43.25	-25.79	-17.46
Br8	C1	-43.28	-25.82	-17.46
Be6	C1	-39.81	-8.56	-31.25
MgBr2–imidazol-2-ylidene				
Mg6	N2	-226.50	-225.95	-0.55
Mg6	N5	-220.35	-219.85	-0.51
Br7	H9	-46.63	-43.78	-2.85
Br7	C1	-43.03	-35.10	-7.94
Br8	H10	-39.51	-38.54	-0.97
Br8	C1	-38.84	-31.81	-7.03
Mg6	C1	19.99	44.85	-24.86
ZnBr2–imidazol-2-ylidene				
Zn6	N2	-145.33	-143.18	-2.15
Zn6	N5	-145.33	-143.18	-2.15
Br7	C1	-41.58	-37.87	-3.71
Br8	C1	-41.58	-37.87	-3.71
Br7	H9	-32.14	-30.48	-1.66
Br8	H10	-32.14	-30.48	-1.66
Zn6	C1	-7.81	73.69	-81.51

Table S3. The most stabilizing IQA atomic pair energies (in kcal/mol) for cyclopropenylidene complexes with MH_2, computed at the MP2/6-311+G(d,p) level of theory. Atom numbering as presented on Fig. S1

Atom A	Atom B	$E_{\mathrm{int}}^{\mathrm{AB}}$	$E_{\mathrm{elst}}^{\mathrm{AB}}$	$E_{\mathrm{ee,XC}}^{\mathrm{AB}}$
BeH2–cyclopropenylidene				
Be6	C1	-201.39	-172.21	-29.19
Be6	C2	-9.66	-9.31	-0.34
Be6	C3	-9.66	-9.31	-0.34
H7	H4	-10.31	-10.29	-0.03
H7	H5	-10.31	-10.29	-0.03
H8	H4	-10.31	-10.29	-0.03
H8	H5	-10.31	-10.29	-0.03
MgH2–cyclopropenylidene				
Mg6	C1	-110.04	-91.23	-18.81
Mg6	C2	-12.48	-12.20	-0.27
Mg6	C3	-12.48	-12.20	-0.27
H7	H4	-8.84	-8.83	-0.01
H7	H5	-8.84	-8.83	-0.01
H8	H4	-8.84	-8.83	-0.01
H8	H5	-8.84	-8.83	-0.01
ZnH2–cyclopropenylidene				
Zn6	C1	-96.34	-30.31	-66.03
Zn6	C2	-7.05	-6.02	-1.03
Zn6	C3	-7.05	-6.02	-1.03
H7	H4	-5.19	-5.18	-0.01
H7	H5	-5.19	-5.18	-0.01
H8	H4	-5.19	-5.18	-0.01
H8	H5	-5.19	-5.18	-0.01

Table S4. The most stabilizing IQA atomic pair energies (in kcal/mol) for cyclopropenylidene complexes with MBr_2, computed at the MP2/6-311+G(d,p) level of theory. Atom numbering as presented on Fig. S1

Atom A	Atom B	$E_{\mathrm{int}}^{\mathrm{AB}}$	$E_{\mathrm{elst}}^{\mathrm{AB}}$	$E_{\mathrm{ee,XC}}^{\mathrm{AB}}$
BeBr2–cyclopropenylidene				
Be6	C1	-219.18	-190.47	-28.71
Br7	H4	-11.14	-11.12	-0.03
Br7	H5	-11.14	-11.12	-0.03
Br8	H4	-11.14	-11.12	-0.03
Br8	H5	-11.14	-11.12	-0.03
MgBr2–cyclopropenylidene				
Mg6	C1	-130.32	-109.02	-21.32
Mg6	C2	-9.38	-9.08	-0.30
Mg6	C3	-9.38	-9.08	-0.30
Br7	H4	-9.72	-9.71	-0.01
Br7	H5	-9.72	-9.71	-0.01
Br8	H4	-9.72	-9.71	-0.01
Br8	H5	-9.72	-9.71	-0.01
ZnBr2–cyclopropenylidene				
Zn6	C1	-112.66	-38.53	-74.13
Zn6	C2	-4.86	-3.76	-1.10
Zn6	C3	-4.86	-3.76	-1.10
Br7	H4	-7.01	-7.00	-0.01
Br7	H5	-7.01	-7.00	-0.01
Br8	H4	-7.01	-7.00	-0.01
Br8	H5	-7.01	-7.00	-0.01

Table S5. The ETS-NOCV–based energy (in kcal/mol) contributions to carbene$\cdots$MX$_2$ bonding. Approximate distortion energies calculated based on the geometries optimized in ωB97X-D/6-311++G**

**MX$_2$**	ΔE_{total}	ΔE_{int}	ΔE_{dist}	ΔE_{elst}	ΔE_{Pauli}	ΔE_{disp}	ΔE_{orb}
			cyclopropenylidene				
BeH_2	-34.44	-45.69	11.25	-69.29	77.03	-2.56	-50.87
MgH_2	-22.20	-26.20	4.00	-45.85	39.55	-2.58	-17.32
ZnH_2	-14.89	-23.63	8.74	-75.38	87.86	-2.87	-33.24
$BeBr_2$	-39.29	-52.23	12.94	-82.67	90.98	-4.90	-55.64
$MgBr_2$	-33.78	-38.57	4.79	-57.23	44.25	-4.14	-21.44
$ZnBr_2$	-28.52	-37.74	9.22	-95.45	104.58	-4.52	-42.34
			imidazol-2-ylidene				
$BeBr_2$	-51.24	-66.86	15.62	-103.25	102.10	-6.61	-59.10
$MgBr_2$	-45.18	-50.49	5.31	-77.00	58.24	-6.01	-25.72
$ZnBr_2$	-41.20	-50.92	9.72	-122.82	128.22	-6.45	-49.87
